# Active Learning Under Expert-Budget Constraints: A Human-in-the-Loop Pipeline for Diabetic Retinopathy Lesion Detection

**DOI:** 10.3390/bioengineering13070762

**Published:** 2026-06-29

**Authors:** Hyeok Kim, Seok-Min Chang, Bo-Young Lim, Soo Young Lee, Ho-Gil Jung

**Affiliations:** 1 Department of Industrial Engineering, Seoul National University of Science and Technology, 232 Gongneung-ro, Nowon-gu, Seoul 01811, Republic of Korea; 19102084@g.seoultech.ac.kr (H.K.); ssk07079@seoultech.ac.kr (S.-M.C.); 2 Department of Data Science, Seoul National University of Science and Technology, 232 Gongneung-ro, Nowon-gu, Seoul 01811, Republic of Korea; esob618@seoultech.ac.kr; 3 Department of Ophthalmology, National Medical Center, Seoul 04564, Republic of Korea; sooyounglee@nmc.or.kr

**Keywords:** diabetic retinopathy, active learning, human-in-the-loop, medical image annotation, YOLOv8, object detection, uncertainty quantification, Monte Carlo dropout

## Abstract

Early diagnosis of Diabetic Retinopathy (DR) is critical for preventing irreversible vision loss, but precise lesion annotation by ophthalmologists is the dominant cost in building any clinical-grade DR detection model. The structural problem in real hospital settings is not labeling cost per se, but expert availability: ophthalmologists’ time is bounded by clinical duties, so the active-learning (AL) cycle can iterate only a handful of times in practice. We frame this constraint explicitly and ask which AL designs work best under a tight expert budget. We propose Virtuous Cycle, a Human-in-the-Loop (HITL) pipeline that integrates (i) a YOLOv8x-based object detector for microaneurysms, hemorrhages, and exudates, (ii) four AL sampling strategies (Average Confidence, Random, Hybrid-Diversity, Monte Carlo Dropout), and (iii) an in-hospital annotation platform (Diavision Studio) in which clinicians refine AI pre-labels rather than draw from scratch. We evaluate Virtuous Cycle on a real-world fundus dataset from the National Medical Center (NMC) across eight AL rounds, expanding the labeled pool from 81 images (R0) to 481 images (R8) within the actual expert-time budget of two ophthalmologists. Across three independent random seeds, random sampling dominates at cold start (mean mAP@50 0.14→0.25 over R0–R1), whereas Hybrid-Diversity converges to the highest mAP@50, Precision, and Recall by R7 (431 images; mAP@50 0.40, Precision 0.55, Recall 0.41), with MC Dropout close behind; by R8, the labeled pool is exhausted and all strategies converge to the same final model. A clinician crossover analysis of 36 paired clinical images, controlling for per-clinician speed bias and per-image difficulty bias, shows no statistically significant difference in overall per-image labeling time between AI-assisted and manual annotation (p=0.52), but a statistically significant increase in confirmed lesion detections under AI assistance (p=0.0058), driven predominantly (84–100% of the net increase) by microaneurysms, the lesion type most prone to being missed unaided. The results indicate that, under expert-budget constraints, AL strategy choice should be staged: random sampling for cold start, uncertainty-and-diversity sampling once the model has matured, and that AI assistance trades a modest, lesion-burden-dependent time cost for a measurable gain in the sensitivity of microaneurysm detection.

## 1. Introduction

Diabetic Retinopathy (DR) is a microvascular complication of diabetes that currently affects more than one hundred million people worldwide and remains a leading cause of adult blindness [1,2]. According to the World Health Organization, the global patient population is projected to exceed 130 million by 2030 [3]. Yet a shortage of ophthalmologists and limited access to screening leave many patients without routine examinations; in Korea, only about half of eligible patients receive early DR screening on schedule [4].

Recent work in deep-learning-based medical artificial intelligence (AI) has demonstrated that automated reading of color fundus images can classify DR severity with accuracy approaching that of specialists [5,6]. Wolf et al. deployed an autonomous AI-based DR screening system in a real clinical trial and raised the screening completion rate to 100% [5], while Dai et al. introduced the DeepDR Plus model, which predicts the five-year risk of DR progression from a single patient’s fundus image [6]. Dow et al. further showed that a two-stage reading workflow combining AI and specialist review achieves a sensitivity of 95.5% and a specificity of 98.2% [7].

Despite these advances, real-world deployment of medical AI still faces three fundamental obstacles: domain shift between training and target hospitals, predictive uncertainty on rare or borderline lesions, and the high cost of acquiring large-scale precisely annotated datasets [8,9,10,11]. To address these issues, the medical-imaging community has converged on two complementary ideas: active learning (AL) [12,13,14], which selects the most informative samples for expert labeling, and human-in-the-loop (HITL) learning [15,16], which keeps clinicians as the decision authority over uncertain model predictions.

### 1.1. The Expert-Budget Bottleneck

On paper, AL and HITL together promise a self-improving “virtuous cycle”: the model proposes labels, the expert corrects them, the corrections become training data, and the next iteration is better. In practice, that cycle is bounded by a quantity the literature rarely names explicitly expert availability. Ophthalmologists in a working hospital cannot label on demand: their time is allocated to clinical duties, and the marginal cost of recalling them for an additional AL round is not trivial. As a result, even a well-engineered AL pipeline only completes a handful of rounds before the expert budget is exhausted. This is qualitatively different from the unconstrained AL regimes studied in benchmark settings [17,18,19], where the next round of acquisitions is essentially free. The right question for hospital deployment is therefore not “can we keep iterating until convergence?” but “which AL strategies make the best use of a small, fixed number of expert-time slices?”

### 1.2. Summary of Identified Gaps

The discussion above reveals three concrete gaps in the existing DR-AI literature: (1) fully autonomous DR readers [5,6,20] maximize throughput but discard the clinician’s marginal expertise, leaving no mechanism for continual model improvement; (2) AI–specialist collaborative workflows [7,21] reintroduce the clinician but lack any sample-selection mechanism, so expert time is spent on whichever images happen to arrive next rather than on the images that would most improve the model; (3) the AL literature in medical imaging [12,14,22,23] concentrates on classification tasks under unconstrained iteration budgets, leaving open the question of whether—and which—AL strategies still work on the harder lesion-detection setting of DR and under a tight expert budget that only permits a handful of rounds.

### 1.3. Our Contributions

The incremental contribution of this study lies in empirically quantifying the trade-off between annotation efficiency and diagnostic recall under strict expert-budget constraints, demonstrating that AI assistance fundamentally shifts the clinical annotation paradigm toward quality enhancement for subtle microvascular lesions rather than simple time reduction. This work makes three contributions:

- **Expert-Budget-Aware AL + HITL Pipeline (Virtuous Cycle).** We design and deploy an AL + HITL pipeline whose query strategy and per-round training hyperparameters are calibrated for a small, fixed number of expert-time rounds rather than for unconstrained iteration. The pipeline integrates a YOLOv8x detector [24], four AL sampling strategies, and an in-hospital annotation platform (Diavision Studio) currently operational on the intranet of the National Medical Center (NMC), Seoul. We surface the expert-budget constraint as a first-class design parameter rather than an implicit limit. This addresses the labeling-cost obstacle highlighted by [8,10].
- **Few-Round AL Convergence Analysis on Clinical DR Lesion Detection.** We provide an 8-round empirical comparison of four AL sampling strategies—Average Confidence, Random Sampling, Hybrid-Diversity, and MC Dropout [25,26]—on real NMC fundus images (pool size R0 = 81 → R8 = 481 labeled images, +50 per round), averaged across three independent random seeds, revealing that Random dominates at cold start while Hybrid (mAP@50 = 0.40 at R7) and MC Dropout (mAP@50 = 0.38 at R7) converge to the highest accuracy before all strategies necessarily collapse to the same final model once the pool is exhausted at R8 (see Section 4.2.1). To our knowledge, this is the first such comparison reported on the object-detection variant of DR, in contrast to existing AL-on-DR work that targets image-level classification only [22], and the round budget reported here reflects what is actually feasible inside one ophthalmology service.
- **Crossover-Validated HITL Labeling-Cost and Quality Analysis with Real Clinicians.** Through a 2 × 2 crossover with two NMC ophthalmologists, with a 36-image-pair log analysis of the deployed Diavision Studio platform (version 1.0), we show that AI-assisted pre-labeling yields no statistically significant change in overall per-image annotation time relative to manual labeling (p=0.52), but a statistically significant increase in confirmed lesion detections (p=0.0058) driven predominantly by microaneurysms—the lesion type clinicians are most prone to miss unaided (Section 4.2.2). The crossover design controls for both per-clinician speed bias and per-image difficulty bias—a measurement discipline absent from prior HITL labeling-cost studies [15].

## 2. Related Work

The literature most relevant to Virtuous Cycle spans four threads: (i) the trajectory of DR-AI from offline benchmarks to deployed autonomous readers, (ii) active learning (AL) as a general framework and as applied to medical imaging, (iii) the much smaller body of work on AL for object detection (as opposed to classification), and (iv) human-in-the-loop (HITL) annotation systems combined with uncertainty quantification (UQ). We discuss each in turn, then position Virtuous Cycle against the closest prior work.

### 2.1. Deep Learning for Diabetic Retinopathy: From Benchmarks to Deployment

The modern era of deep-learning-based DR screening opens with Gulshan et al. [27], who trained a deep convolutional network on 128,175 images and matched the sensitivity and specificity of US-board-certified ophthalmologists on referable DR. Ting et al. [28] extended the result to a multi-ethnic cohort of 494,661 retinal images from the Singapore National Diabetic Retinopathy Screening Program. These two studies established that the recognition problem was solvable; the next decade has been about deployment.

#### 2.1.1. Autonomous AI Readers

Abràmoff et al. [20] conducted the first pivotal clinical trial of an autonomous AI diagnostic system (IDx-DR, now LumineticsCore) in primary-care offices, reporting sensitivity 87.2%, specificity 90.7%, and gradability 96.1% on 900 prospectively screened patients; this trial led to the first US FDA de novo authorization of an autonomous diagnostic AI without clinician interpretation. The EyeArt system, evaluated by Heydon et al. [21] on 30,405 consecutive screening episodes within the English National Diabetic Eye Screening Programme, achieved 95.7% sensitivity for referable retinopathy and obtained CE (Conformité Européenne) marking. Bellemo et al. [29] validated an ensemble deep-learning model in a population-based screening study in Zambia, demonstrating that DR-AI can transfer to under-resourced settings. Wolf et al. [5] subsequently reported the ACCESS randomized control trial, in which an autonomous AI raised the screening completion rate among youth with diabetes to 100%, and Dai et al. [6] introduced DeepDR Plus, which predicts the five-year risk of DR progression from a single fundus image. The common engineering principle in all four works is that the clinician is moved out of the inference loop; throughput and access are maximized at the cost of any continual feedback signal.

#### 2.1.2. AI–Specialist Collaborative Workflows

A parallel line of work argues that for hard or borderline cases, the clinician should remain in the loop. Dow et al. [7] reported a two-stage AI-then-specialist over-read workflow at Stanford, achieving sensitivity 95.5% and specificity 98.2% on referable DR. Gulshan et al. [30] subsequently studied algorithm-vs-manual grading at scale in India and quantified the divergence between automated and human readings on telemedicine workflows. Krause et al. [31] provided the methodological backbone for these studies by quantifying grader variability itself: with majority decision and adjudication-based reference standards, the same algorithmic prediction can shift from “error” to “correct” depending on which expert is asked. This finding is directly relevant to any HITL design: an AL system that selects “uncertain” images for relabeling is, in practice, selecting images on which expert graders themselves disagree.

#### 2.1.3. Public DR Datasets

Outside clinic-internal data, three publicly available datasets dominate the DR-detection literature. Messidor and its successor Messidor-2 [32] provide 1200 posterior-pole fundus images with image-level retinopathy and macular-edema grades. IDRiD [33] is the only widely used dataset with pixel-level lesion annotations for the same four classes used in our NMC dataset (microaneurysms, hemorrhages, soft exudates, hard exudates), making it the most direct external benchmark for lesion-detection methods. The Chinese DDR dataset [34] contributes ∼10,000 images across five ICDR severity levels and supports both classification and lesion-segmentation tasks. None of these datasets, however, capture the iterative-annotation regime our work targets: they are pre-collected and frozen, whereas a deployed AL + HITL system has to acquire labels online.

#### 2.1.4. Limitations Identified by Recent Surveys

Two persistent obstacles are highlighted by recent DR-AI reviews: domain shift between training and target hospitals and the prohibitive cost of precise annotation [8]. Reviews of medical-image deep learning more broadly [10,11] identify the same annotation bottleneck as the single most cited obstacle to clinical translation. Lambert et al. [9] note in addition that any clinically deployed AI needs principled uncertainty quantification before clinicians will trust its outputs—a point that re-enters our discussion in Section 2.4.

### 2.2. Active Learning: Theory and Application to Medical Imaging

Active learning has a long methodological lineage; we draw from both the general AL literature and its medical-imaging specialization.

#### 2.2.1. Foundational AL Strategies

Settles [13] surveys the classical AL landscape—pool-based vs. stream-based querying, uncertainty sampling, query-by-committee, expected-model-change, and density-weighted variants—and remains the standard reference. Houlsby et al. [35] introduced Bayesian Active Learning by Disagreement (BALD), which selects samples that maximize mutual information between predictions and model parameters; in modern implementations, BALD is operationalized via MC Dropout [25], the same Bayesian-approximation principle we use in our MC Dropout strategy. Sener and Savarese [17] reformulated AL as a core-set selection problem on deep-network feature embeddings, providing a geometric guarantee on the gap between the selected subset and the full pool. Yoo and Kweon [19] proposed a task-agnostic loss-prediction module that estimates the expected loss of unlabeled images and queries those with the largest predicted loss. Kirsch et al. [18] extended BALD to the realistic batch-acquisition setting, showing that naive per-sample BALD acquires redundant points and that mutual information among the acquired batch must be jointly maximized. These four works (core-set, loss-prediction, BALD, and BatchBALD) define the modern AL toolbox.

#### 2.2.2. AL in Medical Imaging

Specializing in medical imaging, Budd et al. [14] provide a focused survey of AL and HITL deep learning, arguing that the unique constraints of medical AI (expert sparsity, regulatory burden, longitudinal patient data) demand that the human stay in the loop. Wang et al. [12] update the picture with a comprehensive 2024 survey covering both classification and segmentation. Gaillochet et al. [36] introduced stochastic-batch AL for 3D MRI segmentation, computing uncertainty at the batch level rather than per sample and combining diversity with informativeness—conceptually similar to the Hybrid-Diversity strategy we evaluate. Ma et al. [23] proposed adaptive-curriculum AL, in which the query order shifts from diversity (cold start) towards uncertainty (mature model) as training progresses; we adopt the same conceptual shift but instantiate it operationally as switching among four different strategies across rounds.

#### 2.2.3. AL Specifically on Diabetic Retinopathy

Within DR, Paul, Pan, and Sobol [22] compared four deep-AL acquisition functions (entropy, BALD, core-set, adversarial) on 88,702 retinal fundus images and showed that a small fraction of labels suffices to approach fully supervised accuracy. Their evaluation is at the image classification level (DR present/absent/severity grade), not at the bounding-box detection level. The transferability of these results from classification to lesion detection has, to our knowledge, not been studied—which is the gap our work addresses.

### 2.3. Active Learning for Object Detection

AL for object detection is substantially harder than AL for classification, because per-sample uncertainty is no longer a single softmax entropy: each image contains a variable number of predicted bounding boxes, each with its own class score and localization confidence, and an image-level acquisition score must aggregate across these. Yuan et al. [37] address this with Multiple Instance Active Object Detection (MI-AOD), which treats each image as an instance bag and learns instance-level uncertainty through two adversarial classifiers, producing image-level acquisition scores via multiple-instance learning. Yu et al. [38] introduced Consistency-based Active Learning for object Detection (CALD), which exploits consistency between predictions on original and augmented versions of the same image to define a per-image acquisition score and adds a mutual-information term to encourage balanced class distributions; on Faster R-CNN over PASCAL VOC and MS COCO, CALD outperformed random sampling by 0.8–2.9 mAP.

Neither MI-AOD nor CALD has been evaluated on medical lesion detection. Our work fills part of that gap by evaluating four AL strategies (one uncertainty-only, one diversity-only, one hybrid, one MC Dropout-based) on a real clinical lesion-detection task with bounding-box labels. The four strategies are conceptually simpler than MI-AOD or CALD, which is a deliberate choice: under the expert-budget constraint described in Section 1, strategies that require additional model components (instance classifiers, augmentation-consistency heads) increase the per-round engineering cost and reduce the number of round iterations a hospital team can realistically run.

### 2.4. Human-in-the-Loop, Uncertainty, and Annotation-Cost Studies

#### 2.4.1. HITL in Clinical AI

HITL approaches treat the clinician as an active component of the learning system rather than as a one-off annotator. Vásquez-Venegas et al. [15] applied a HITL design to COVID-19 lung-CT segmentation, iterating model retraining with radiologist feedback for three cycles and improving Dice by 0.2–0.5 over the baseline. Yalcinkaya et al. [16] developed a temporal uncertainty-localization metric for cardiac-MRI time-series segmentation that automatically flags segments warranting expert review—an early example of an HITL system that uses model uncertainty to triage clinician attention rather than asking the clinician to review every output.

#### 2.4.2. Uncertainty Quantification for HITL Triage

The HITL designs above all depend on a reliable per-image uncertainty signal. Gal and Ghahramani [25] showed that dropout at inference time approximates Bayesian inference, enabling cheap per-sample uncertainty estimates without modifying the training procedure—the foundation of our MC Dropout sampling strategy. Kendall and Gal [26] separated aleatoric uncertainty (inherent in the data) from epistemic uncertainty (model-resolvable with more data), and argued that AL is principally interested in the latter. Lakshminarayanan et al. [39] proposed deep ensembles as a simple, scalable alternative to MC Dropout for uncertainty estimation. Lambert et al. [9] review the entire UQ-for-medical-imaging field and argue that clinically deployable systems must surface uncertainty in a form clinicians can act on, not merely measure it.

#### 2.4.3. Annotation-Cost Reviews

Quantitative studies of annotation cost in medical imaging form a small but growing literature. Cheplygina, de Bruijne, and Pluim [11] survey 140+ works on semi-supervised, multi-instance, and transfer learning in medical imaging, identifying labeled-data scarcity as the central design driver. Tajbakhsh et al. [10] extend this with a review of deep-learning solutions for medical segmentation under imperfect datasets: scarce annotations, weak annotations, and noisy annotations. Both reviews argue that the field has under-invested in measurement discipline: per-image labeling time is rarely reported, std-dev across labelers is rarely reported, and the cumulative cost of one full annotation pass is almost never reported in comparable units. Our crossover labeling-time experiment in Section 4.2.2 is one small attempt at the discipline these reviews call for.

#### 2.4.4. Open Issues

Together, the HITL and UQ literature show that AL and HITL can improve both model reliability and labeling efficiency under tight data budgets. However, two open issues remain. First, most HITL studies are short-horizon experiments with ≤3 retraining cycles, and longitudinal integration of HITL into routine clinical workflows is under-studied [9,14]. Second, the practical budget on expert-time—as opposed to compute or data—is rarely treated as a first-class design variable, despite being the most binding constraint in deployed clinical settings. Virtuous Cycle is designed around exactly this constraint.

### 2.5. Positioning: How Virtuous Cycle Differs

The literature above intersects Virtuous Cycle on four axes—DR-AI deployment, active learning, AL for object detection, and HITL + UQ—but no prior system combines all four. Table 1 summarizes the distinction.

Concretely, Virtuous Cycle is distinct on the following points:

- **Against autonomous DR readers (Wolf [5], Dai [6], Abràmoff [20], Heydon [21], Bellemo [29]).** All four maximize throughput by removing the clinician from the inference loop; Virtuous Cycle keeps the clinician as the decision authority and turns every correction into a new training sample. The trade-off is throughput vs. continual improvement; Virtuous Cycle chooses the latter.
- **Against AI–specialist collaborative workflows (Dow [7], Krause [31]).** Dow et al. confirm that specialist over-read of AI output improves sensitivity and specificity, but their workflow has no sample-selection mechanism: the specialist over-reads whichever images happen to arrive. Krause et al. further show that “which expert is asked” itself moves the reference standard. Virtuous Cycle adds active learning on top of these designs so that the specialist’s marginal effort is targeted at the most informative samples.
- **Against active-learning-on-DR work (Paul [22]).** Paul et al. compare four deep-AL strategies on DR but at the image-classification level. Virtuous Cycle evaluates the same family of strategies on the strictly harder object-detection task (bounding boxes around MA, HE, and EX lesions), where the per-sample uncertainty signal must be aggregated across multiple boxes per image rather than read off a single softmax.
- **Against AL-for-detection methods (MI-AOD [37], CALD [38]).** Both methods are evaluated on PASCAL VOC/MS COCO with simulated labelers and unconstrained round budgets. Virtuous Cycle evaluates simpler strategies on a real medical imaging task with a real (and binding) expert budget. The trade-off is acquisition sophistication vs. engineering simplicity per round; under tight expert budgets, simplicity wins.
- **Against adaptive AL methods (Ma [23]).** Ma et al. propose curriculum-based AL on generic classification benchmarks. Virtuous Cycle’s adaptation is operational rather than curriculum-based: we adjust both the sampling strategy (random for cold start, hybrid/MC dropout for mature models) and the YOLOv8x per-round hyperparameters as the labeled pool grows, and we validate the adaptation in the deployed clinical workflow.
- **Against HITL studies in other modalities (Vásquez-Venegas [15], Yalcinkaya [16]).** These works keep the clinician in the loop on CT and cardiac-MRI tasks; Virtuous Cycle ports the principle to color fundus lesion detection and additionally quantifies the per-image speed-up under a 2×2 crossover that controls for both clinician identity and image difficulty—a measurement discipline that prior HITL labeling-cost studies have not applied.
- **Against UQ foundations (Gal–Ghahramani [25], Kendall–Gal [26], Lakshminarayanan [39], BALD [35], BatchBALD [18]).** Virtuous Cycle does not propose a new UQ method; we instantiate MC Dropout (the cheapest of these for a deployed detector) and study how the resulting uncertainty signal interacts with annotation throughput under the expert-budget constraint.

To our knowledge, Virtuous Cycle is the first system to simultaneously (a) operate on object-detection-level DR labels (rather than image-level), (b) compare four AL strategies in a single hospital deployment, (c) surface the expert-budget constraint as a first-class design parameter, and (d) report HITL labeling-cost reduction from real clinicians under a controlled crossover.

## 3. Methodology

### 3.1. Systematic Overview

**Figure 1:**
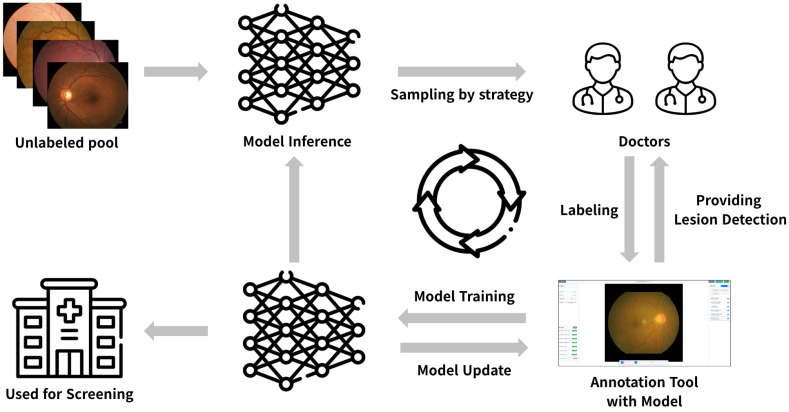
Overall framework architecture.

Figure 1 illustrates the overall architecture, Virtuous Cycle, proposed in this study. The proposed pipeline integrates active learning and HITL labeling into a cyclical framework. Initially, a YOLOv8x-based object detection model [24] is trained using a small subset of manually labeled data. Subsequently, the trained model actively selects highly informative samples from an unlabeled dataset based on specific strategies and requests expert labeling. Physicians then review and refine the model’s predictions via an integrated annotation platform to generate final labels, which are appended to the training dataset. Retraining the model with this updated data improves its performance, and this iterative process progressively refines the model. The key steps are outlined as follows:

1. **Model Initialization:** The YOLOv8x object detector is initially trained on a small number of fundus images using expert-provided bounding box annotations.
2. **Active Learning Sampling:** The system selects the next batch of unlabeled images using one of four sampling strategies (Section 3.3); strategies are chosen per round to balance uncertainty and data diversity.
3. **Human-in-the-Loop Labeling:** Ophthalmologists perform annotations on the platform, utilizing the AI-generated predictions as initial drafts.
4. **Model Update:** The model is retrained with the newly acquired labels to enhance detection performance.

Through this cycle, the proposed framework maximizes model accuracy while minimizing annotation costs subject to the expert-budget constraint described in Section 1. The initial model in this study was trained on a cold-start pool of 81 high-quality fundus images from the National Medical Center (NMC) dataset; the pool was expanded to 481 cumulative images over eight rounds of active learning (50 acquisitions per round; see Section 4). Algorithm 1 states the outer loop formally; the sampling step (line 3 of Algorithm 1) is detailed by Algorithm 2 in Section 3.3.
**Algorithm 1** Virtuous Cycle—Expert-Budget-Aware AL + HITL Round Loop**Require:** Initial labeled set $L_{0}$; unlabeled pool $U_{0}$; number of rounds *R*; per-round acquisition budget *k*; strategy schedule σ:{0,…,R}→ {Rand, AvgC, Hyb, MCD}; training hyperparameter schedule θ:{0,…,R}→Θ (Table 2 and Table 3); clinicians E available for HITL review.**Ensure:** Trained detector $f_{R}$ and labeled corpus $L_{R}$.  1:*f*_0_ ← Tranin$\left(L_{0},\;\theta \left(0\right)\right)$                                          ▹ cold start on $|L_{0}|\;=\;81$ images  2:**for** r = 1 **to** *R* **do**  3:    $S_{r}\leftarrow$ SamplingStrategy $\left(\sigma \left(r\right),\;f_{r-1},\;U_{r-1},\;k\right)$                                 ▹ Algorithm 2  4:    ${\tilde{Y}}_{r}\leftarrow \left\{\;f_{r-1}\left(x\right):x\in S_{r}\;\right\}$                  ▹ AI pre-labels surfaced in Diavision Studio  5:    $Y_{r}\leftarrow$ ExpertReview $\left(S_{r},\;{\tilde{Y}}_{r},\;E\right)$                  ▹ HITL: clinician edits the pre-labels  6:    $L_{r}\leftarrow L_{r-1}\cup \left\{\left(x,y\right):x\in S_{r},\;y\in Y_{r}\right\}$  7:    $U_{r}\leftarrow U_{r-1}\setminus S_{r}$  8:    $f_{r}\leftarrow$ Train $\left(L_{r},\;\theta \left(r\right)\right)$                  ▹ hyperparameters adapt with $|L_{r}|$ per Table 3  9:**end for**10:**return** $f_{R},\;L_{R}$

**Table 2:** Training hyperparameters for YOLOv8x.

Parameter	Value	Description
imgsz	1280	Input image size
optimizer	AdamW	Optimizer
final learning rate	0.01	Final learning-rate factor (${\mathrm{lr}}_{0}\times {\mathrm{lr}}_{f}$)
momentum	0.937	SGD/Adam momentum
weight_decay	0.0005	Weight-decay coefficient
hsv_h	0.005	HSV hue adjustment range
hsv_s	0.1	HSV saturation adjustment range
hsv_v	0.1	HSV value adjustment range
degrees	3	Random rotation range (±3°)
translate	0.03	Image translation range (±3%)
scale	0.2	Image scaling range (±20%)
flipud	0.0	Vertical flip probability (disabled)
fliplr	0.5	Horizontal flip probability
amp	True	Automatic mixed precision

**Table 3:** Adaptive Hyperparameters by Round Number.

Parameter	Initial (round_num < 2)	Later Rounds (round_num > 2)
epochs	80 + round_num × 10	80 + round_num × 10
batch	4	8
patience	20	30
learning rate	0.002	0.0025
mosaic	0.2	0.3
close_mosaic	40	60

Two design choices in Algorithm 1 are direct consequences of the expert-budget framing: *R* is small and fixed (we use R=8, the budget two NMC ophthalmologists could realistically devote across the deployment period), and the strategy schedule σ is allowed to switch across rounds rather than being held constant—a key empirical finding in Section 4.2.1 is that no single strategy dominates throughout, so the schedule should drift from Random at r=0,1 toward Hybrid-Diversity/MC Dropout at later rounds.

### 3.2. Object Detection Model

At the core of the proposed pipeline lies the YOLOv8x object-detection model [24]. With its strong backbone network, YOLOv8x efficiently detects a range of abnormalities, including microvascular lesions, in complex retinal images. The model is chosen for its trade-off between near-real-time inference and high detection accuracy on high-resolution medical images. During training, active learning starts from pre-trained weights obtained on standard object-detection datasets, and we tune the hyperparameters empirically to ensure stable convergence. Table 2 and Table 3 list the configurations used. The parameters in Table 3 are adjusted across rounds to track the size of the labeled pool. All four AL strategies share the identical round-*r* hyperparameter configuration in Table 3 at every round *r*; the round-to-round schedule (epochs, batch size, mosaic augmentation) is a function of the labeled-pool size only, regardless of which sampling strategy produced that pool, so that any two strategies compared at the same round are trained under strictly identical settings.

### 3.3. Sampling Strategy in Active Learning

During the active-learning phase, information-based sampling chooses which unlabeled images to send for expert review, with the aim of maximizing model improvement under the per-round acquisition budget *k*. We implement and compare four strategies.

#### Notation

Let X be the pool of unlabeled fundus images and k∈N be the per-round acquisition budget (in this work, k=50). For an input image x∈X, let B(x) be the set of bounding boxes predicted by the YOLOv8x model, N=|B(x)|, and let conf(b) denote the (softmax-normalized) confidence score of box *b*. We write $\bar{\mathrm{conf}}\left(x\right)=\frac{1}{N}\sum_{b\in B(x)}\mathrm{conf}\left(b\right)$ for the image-level mean confidence. The operators bottom-k and top-k return the subset of X whose per-image scores are the *k* smallest/largest, breaking ties uniformly at random. All set unions below are interpreted as set unions (not multisets) and we enforce disjointness by drawing the two component sets without replacement from a shared pool.

1. Average Confidence: Prioritizes images with a low average confidence score across all predicted bounding boxes. This instantiates the classical least-confidence/uncertainty-sampling paradigm [13,40]:
  (1) $$s_{A}\left(x\right):=\bar{\mathrm{conf}}\left(x\right),\;S_{A}=\mathrm{bottom}\text{-}k\left(s_{A}\right),$$
  where $S_{A}$ denotes the subset of images selected by choosing the *k* samples with the lowest (bottom-*k*) average confidence scores.
2. Random Sampling: Serves as a comparative baseline where all images are selected uniformly at random. This approach is advantageous for securing diverse data during the initial stages:
  (2) $$S_{B}∼i.i.d.\;\mathrm{Uniform}\left(X\right),\;\left|S_{B}\right|=k\;(\mathrm{sampled}\;\mathrm{without}\;\mathrm{replacement}),$$
  where X denotes the entire pool of unlabeled datasets available for sampling.
3. Hybrid-Diversity: Selects samples from highly uncertain images while preserving data diversity; conceptually related to the diversity-plus-informativeness combination of Gaillochet et al. [36] and the diversity-to-uncertainty curriculum of Ma et al. [23] discussed in Section 2.2. Let ρ∈(0,1) be the uncertainty fraction; in our experiments ρ=0.7, selected empirically during preliminary tests to provide the optimal balance between exploiting uncertainty and maintaining data diversity within our limited per-round batch size. We first select the ρk images with the lowest mean confidence, then complete the budget with random draws disjoint from $S_{\mathrm{unc}}$:
  (3) $$\begin{array}{cc} S_{\mathrm{unc}} & =\mathrm{bottom}\text{-}\left(\rho k\right)\left(\bar{\mathrm{conf}}\right),\;S_{\mathrm{rand}}=\mathrm{Sample}\left(X\setminus S_{\mathrm{unc}},\;\left(1-\rho \right)k\right), \end{array}$$
  (4) $$\begin{array}{cc} S_{C} & =S_{\mathrm{unc}}\cup S_{\mathrm{rand}},\;\left|S_{C}\right|=k. \end{array}$$
  Because $S_{\mathrm{rand}}$ is sampled from $X\setminus S_{\mathrm{unc}}$, the two component sets are disjoint by construction and $\left|S_{C}\right|=\rho k+\left(1-\rho \right)k=k$ holds exactly. This balances exploring uncertain decision boundaries with maintaining global data diversity.
4. Monte Carlo Dropout: Following Gal and Ghahramani [25], we keep dropout active during inference and run *T* stochastic forward passes per image (we set T=10, following the standard range used by Gal and Ghahramani [25] for efficient epistemic-uncertainty estimation). Let $p^{\left(t\right)}\left(x\right)\in {\left[0,1\right]}^{C}$ be the per-class detection-confidence vector produced by the *t*-th pass, i.e., ${\left[0,1\right]}^{C}$ denotes the *C*-dimensional unit hypercube in which each entry is the softmax probability for one of the *C* lesion classes, where *C* is the number of lesion classes (MA, HE, EX); $p_{c}^{\left(t\right)}\left(x\right)$ denotes the *c*-th scalar entry of $p^{\left(t\right)}\left(x\right)$, i.e., the confidence specifically for class *c*. We combine a low-confidence term with an epistemic-uncertainty term:  
  (5) $$\begin{array}{cc} u(x) & =\frac{1}{C}\sum_{c=1}^{C}{\mathrm{Var}}_{t}\;\left(p_{c}^{\left(t\right)}\left(x\right)\right), \end{array}$$
  (6) $$\begin{array}{cc} s_{D}\left(x\right) & =\lambda \left(1-\bar{\mathrm{conf}}\left(x\right)\right)\;+\;\left(1-\lambda \right)\;u\left(x\right), \end{array}$$
  (7) $$\begin{array}{cc} S_{D} & =\mathrm{top}\text{-}k(s_{D}), \end{array}$$
  where ${\mathrm{Var}}_{t}\left(\cdot \right)$ is the empirical variance across the *T* stochastic passes, computed per class and then averaged over the *C* classes; λ∈(0,1) is the convex-combination weight (set to the same value as ρ in our experiments; both ρ and λ were chosen empirically during preliminary tests to balance the diversity and uncertainty terms within the limited per-round batch size, rather than via a systematic sweep). The score u(x) quantifies epistemic uncertainty in the sense of approximate Bayesian inference via MC Dropout [25]; the final subset $S_{D}$ is the top-*k* images by combined score $s_{D}\left(x\right)$. We use the symbol λ here, rather than α, to avoid clashing with the Hybrid-Diversity fraction ρ introduced above.

Each strategy selects informative samples based on uncertainty, diversity, or both, thereby reducing redundant labeling effort. Prior medical-imaging studies show that such active-learning regimes can achieve accuracy comparable to fully supervised training while using only a fraction of the available training data [12,22,36].

Algorithm 2 states the four strategies as a single routine indexed by the strategy enum σ∈ {AvgC, Rand,Hyb,MCD} that Algorithm 1 hands in at every round. Notation follows the Notation Section above.
**Algorithm 2** SamplingStrategy—unified acquisition for the four AL strategies**Require:** Strategy σ∈{AvgC, Rand, Hyb, MCD}; current detector *f*; unlabeled pool X; acquisition budget *k*; hybrid fraction ρ∈(0,1); MC Dropout passes T∈N; MC Dropout weight λ∈(0,1); number of lesion classes *C*.**Ensure:** S⊆X with |S|=k.  1:**For each** x∈X: compute predicted boxes B(x)←f(x) and the per-image mean confidence $\bar{\mathrm{conf}}\left(x\right)\leftarrow \frac{1}{\left|B(x)\right|}\sum_{b\in B(x)}\mathrm{conf}\left(b\right)$.  2:**if**σ= AvgC **then**                                                                                                ▹ Average-Confidence  3:    $S\leftarrow \mathrm{bottom}\text{-}k\left(\bar{\mathrm{conf}}\right)$  4:**else if** σ=  Rand **then**                                                                         ▹ uniform Random sampling  5:    S← AmpleWithoutReplacement(X,k)  6:**else if** σ=  Hyb **then**                                                                                            ▹ Hybrid-Diversity  7:    $S_{\mathrm{unc}}\leftarrow \mathrm{bottom}\text{-}\left(\rho k\right)\left(\bar{\mathrm{conf}}\right)$  8:    $S_{\mathrm{rand}}\leftarrow$ AmpleWithoutReplacement$\left(X\setminus S_{\mathrm{unc}},\;\left(1-\rho \right)k\right)$  9:    $S\leftarrow S_{\mathrm{unc}}\cup S_{\mathrm{rand}}$                                                                            ▹ disjoint by construction; |S|=k10:**else if** σ= MCD **then**                                                                                    ▹ Monte-Carlo Dropout11:    **for** x∈X **do**12:        **for** t=1 **to** *T* **do**13:           $p^{\left(t\right)}\left(x\right)\leftarrow f_{\mathrm{dropout}}\left(x\right)$                                                            ▹ dropout kept active at inference14:        **end for**15:        $u\left(x\right)\leftarrow \frac{1}{C}\sum_{c=1}^{C}{\mathrm{Var}}_{t}\;\left(p_{c}^{\left(t\right)}\left(x\right)\right)$16:        $s_{D}\left(x\right)\leftarrow \lambda \;\left(1-\bar{\mathrm{conf}}\left(x\right)\right)+\left(1-\lambda \right)\;u\left(x\right)$17:    **end for**18:    $S\leftarrow \mathrm{top}\text{-}k(s_{D})$19:**end if**20:**return** S

Two implementation notes. First, the *T* inner loop of the MCD branch dominates the per-round wall-clock cost (T=10 multiplies inference time by an order of magnitude); when the per-round expert budget is tight, this cost is acceptable because the expert work that follows is far slower than the AI inference. Second, the Rand branch is a strict generalization-vs-bias baseline rather than a degenerate case: as shown in Section 4.2.1, it dominates the other three strategies in the cold-start regime (R0→R1) and is the strategy of choice at r=0.

### 3.4. HITL Platform

**Figure 2:**
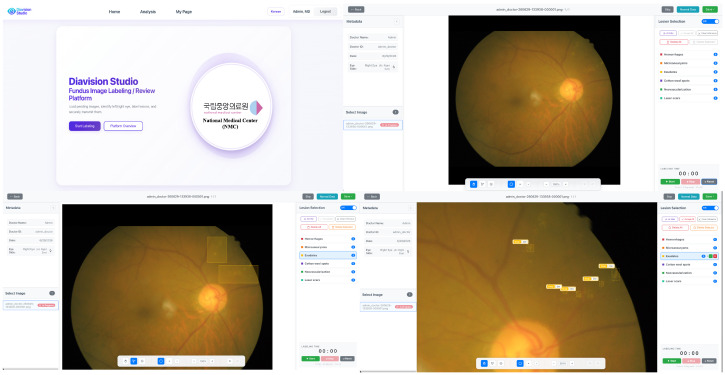
HITL Annotation Platform Used at NMC.

Figure 2 illustrates the “Diavision Studio” service, which is currently deployed on the NMC intranet in South Korea. For the annotation phase, we developed a HITL annotation platform that operates securely within the hospital intranet environment. To tailor the system to clinical use, multiple rounds of user experience (UX) interviews with ophthalmologists, feedback sessions, and quality assurance tests were conducted.

The platform is designed to allow physicians to compare and refine AI-generated predictive labels against manual annotations within a single, unified interface. It features an intuitive, keyboard-shortcut-driven workflow, along with version control and logging capabilities, optimized to accelerate the expert annotation process while ensuring high-quality standards. Notably, by automatically presenting AI-generated pre-labels, the system enables physicians to finalize accurate annotations with only minor adjustments, thereby significantly reducing both the time and cost associated with the labeling process.

## 4. Performance Evaluation

### 4.1. Dataset

To evaluate the effectiveness of the object-detection model in reducing lesion-annotation time in a real-world clinical setting, this study uses the NMC dataset, made available under official NMC approval and comprising three lesion classes: microaneurysms (MA), hemorrhages (HE), and exudates (EX). The dataset is curated by ophthalmologists Dr. Ho-Gil Jung and Dr. Soo Young Lee of the NMC using the Diavision Studio platform. Although the clinical inference system itself is beyond the scope of this experiment, the annotation framework described here is in clinical use at the NMC, and the labels collected during routine reading continue to feed model updates. The same held-out test set is used to evaluate every active-learning round (R0 through R8) and the final converged model, ensuring a fair, round-invariant comparison across strategies.

### 4.2. Experimental Results

#### 4.2.1. Model Performance Convergence Analysis Based on Sampling Strategy

**Figure 3:**
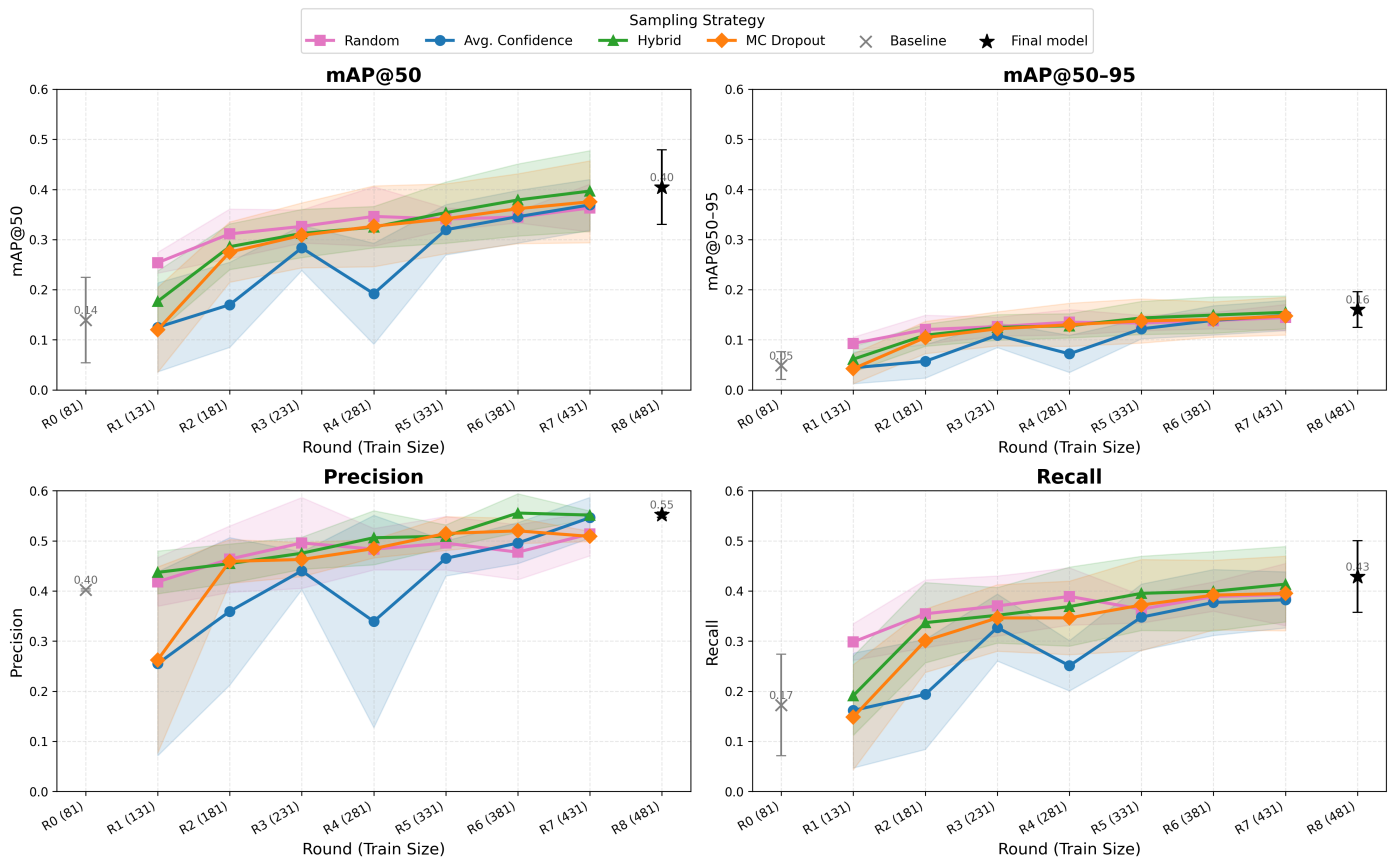
Model performance (mean ± std across three independent random seeds) across active-learning rounds for the four sampling strategies on the NMC dataset. Metrics: mAP@50, mAP@50–95, Precision, and Recall. Hybrid and, to a lesser extent, MC Dropout outperform Random and Average Confidence sampling in later rounds; all strategies converge at R8 once the labeled pool is exhausted.

We analyze the performance differences among the four active-learning sampling strategies (Average Confidence, Random Sampling, Hybrid-Diversity, and MC Dropout) on the NMC dataset. Each strategy starts from the cold-start pool of 81 images (R0) and acquires 50 additional samples per round. The model is retrained iteratively across R=8 rounds indexed r=0,…,8, so the labeled pool grows from 81 at R0 to 431 at R7 and to 81+8×50=481 at R8. Because R8 covers the entire available (non-test) NMC pool, all four strategies necessarily converge to the same final model at R8, regardless of the sampling sequence. We therefore report this converged performance in the R8 column of Table 4 as the reference baseline. To distinguish genuine strategy effects from single-seed variance, we repeated the full 8-round pipeline across three independent random seeds and report mean ± std at every round in Table 4 and Figure 3. The experimental results, summarized in Figure 3, are compared on four representative metrics: mAP@50, mAP@50–95, Precision, and Recall. Detailed per-strategy numerical results are reported in Table 4.

For mAP@50, Random shows the fastest cold-start improvement (mean 0.14 to 0.25 over R0–R1). As demonstrated by the wide standard deviation bands in Figure 3, the model is severely underfitted at the cold-start phase (R1). Consequently, its predicted confidences and MC Dropout variances are highly unstable, rendering uncertainty-based sampling less effective than random sampling during the initial rounds. In later rounds, Hybrid consistently overtakes the other three, reaching a mean of 0.40±0.08 at R7, with MC Dropout close behind at 0.38±0.08; a paired *t*-test across the three seeds confirms Hybrid significantly outperforms MC Dropout at R7 (t=24.4, p=0.0017), though the comparison against Random did not reach significance at this seed count (t=1.45, p=0.28).

For Precision, Hybrid achieves the highest final-round Precision (0.55±0.01 at R7), with Average Confidence close behind (0.55±0.04) and Random and MC Dropout both around 0.51 (the Hybrid-vs-MC Dropout difference is marginal at this seed count: p=0.069). The result is consistent with the hypothesis that uncertainty-aware sampling reduces false-positive detections. For Recall, Hybrid converges most rapidly, reaching a mean of 0.41±0.08 in the R7 round (p=0.098 vs. MC Dropout) and confirming its capacity to detect lesions without omission.

The overall pattern is clear: Random is efficient in the cold-start regime, but transitioning to Hybrid or MC Dropout as the model matures accelerates convergence and improves the final detection metrics, though with the modest seed-to-seed variance reported above, the strategy ranking among Hybrid, MC Dropout, and Average Confidence should be read as a trend rather than a sharply significant ordering at every metric. This is the empirical basis for the round-switchable schedule σ in Algorithm 1: in the deployed system, Random and Hybrid are used in the initial rounds, while MC Dropout is used from the mid- to late-rounds.

#### 4.2.2. Effect of AI-Assisted Labeling System on Reducing Labeling Time

**Figure 4:**
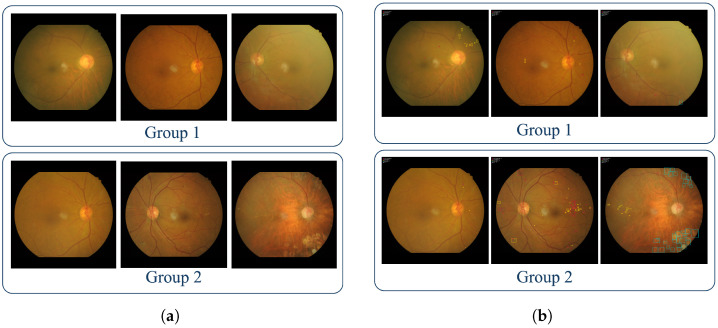
Visualization of the AI-assisted annotation workflow on diabetic-retinopathy images: (**a**) expert-annotated lesions (Dr. Ho-Gil Jung and Dr. Soo Young Lee); (**b**) model-predicted bounding boxes over the same fundus image.

To assess the impact of AI assistance on annotation efficiency and quality, we conducted a statistical analysis of the full annotation logs from the deployed Diavision Studio platform. This analysis comprises a total of 36 image-level AI-vs-Default labeling pairs (31 lesion-containing + 5 lesion-free images). The experiment utilized a 2×2 crossover design to isolate the effect of AI assistance from per-clinician speed bias and per-image difficulty bias; specifically, for any given image, the AI-assisted and manual sessions were performed by different clinicians to avoid same-clinician relabeling or memory bias. Figure 4 illustrates the representative workflow of this crossover design, where images were divided into two groups (Group 1 and Group 2), and two NMC ophthalmologists (Dr. Ho-Gil Jung and Dr. Soo Young Lee) performed annotations as follows: Dr. Jung annotated Group 1 with the AI-assisted system and Group 2 manually, while Dr. Lee annotated Group 1 manually and Group 2 with the AI-assisted system.

**Figure 5:**
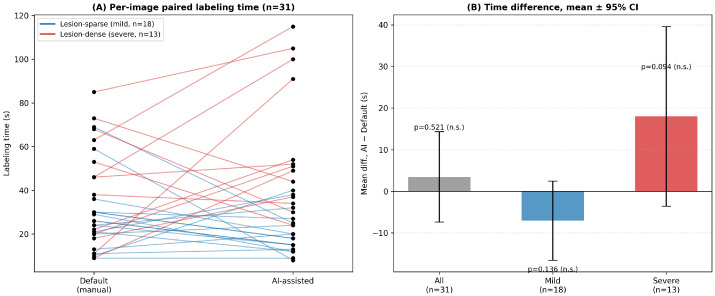
Comparison of per-image annotation time with and without AI assistance. (**A**) Paired per-image labeling time, colored by lesion burden (mild = lesion-sparse, severe = lesion-dense). (**B**) Mean difference (AI-Default) with 95% confidence intervals for the pooled sample and for the mild/severe subgroups; none of the three differences reach statistical significance at this sample size.

A paired *t*-test on this sample shows no statistically significant difference in mean per-image labeling time (Figure 5B; t=0.65, p=0.52; 95% CI for the mean difference: [−7.4s,+14.3s]; Wilcoxon signed-rank p=0.70), indicating that the AI-assisted workflow does not significantly alter overall annotation speed. Stratifying the same sample by lesion burden (using the number of lesion boxes as a severity proxy, split at the median) suggests a crossover pattern: AI assistance trends faster on lesion-sparse images (n=18, −7.1s) and trends slower on lesion-dense images (n=13, +18.0s), but neither subgroup difference reaches significance at this sample size (p=0.136 and p=0.094, respectively); we report this as a directional, hypothesis-generating observation rather than a confirmed effect, and flag the subgroup sample size as a limitation (Section 5).

**Figure 6:**
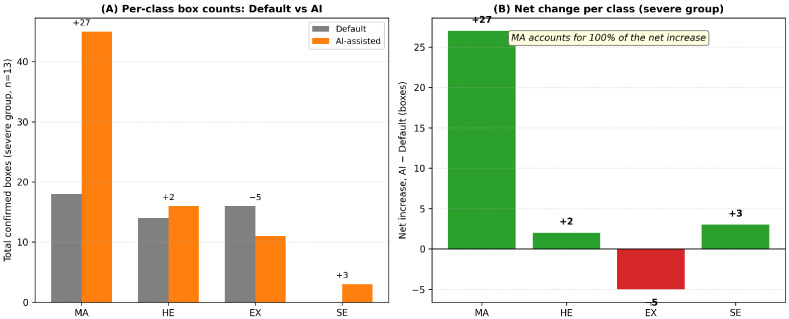
Class-level breakdown of the additional lesion boxes confirmed under AI assistance, within the lesion-dense (severe) subgroup of the 36-pair HITL log analysis. (**A**) Total confirmed boxes per class, Default vs. AI-assisted. (**B**) Net change per class (AI−Default); microaneurysms account for the entire net increase.

Critically, the additional time on lesion-dense images is not wasted; AI assistance significantly increases the total number of confirmed lesion boxes (paired *t*-test, p=0.0058 across the 36-pair sample). Figure 6 decomposes this net increase by class: microaneurysms account for 84–100% of the increase, while exudate counts slightly decreased, indicating that AI assistance surfaces additional instances of the lesion type clinicians are most prone to miss unaided. Repeating the same comparison on lesion-sparse images shows no significant difference (p=0.187).

**Figure 7:**
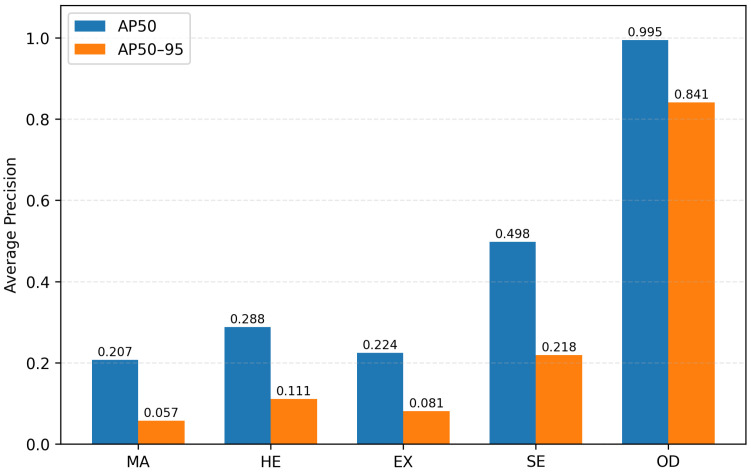
Per-class AP (AP50 and AP50–95) of the multi-class YOLOv8x detector (MA, HE, EX, SE, OD) evaluated on the held-out IDRiD validation split (n=27 images).

A potential concern in object detection is that aggregate metrics like mAP might be dominated by ‘easy’ classes. To verify the robustness of our performance, we analyzed class-specific difficulty using a multi-class YOLOv8x detector evaluated on a held-out IDRiD validation split (Figure 7). The results show AP50 (MA) =0.207, AP50 (HE) =0.288, and AP50 (EX) =0.224. Since microaneurysms (the most clinically significant lesion) are indeed the most challenging class, these results confirm that our aggregate mAP (Table 4) is not artificially inflated by easier lesion types.

These findings indicate that integrating AI assistance into the clinical annotation workflow does not shorten annotation time but shifts clinicians’ effort toward verifying clinically important microaneurysm detections. This reframes the system’s value proposition from time savings to annotation quality enhancement, particularly on lesion-dense images.

## 5. Discussions

The empirical evidence presented in Section 4 demonstrates that integrating active learning with a HITL annotation platform substantially reduces the labeling cost of constructing a clinically usable DR detection model. Nevertheless, several limitations remain that bound the generalizability of the present results.

First, the dataset is acquired with a single fundus camera at a single institution (the National Medical Center, Seoul). As a consequence, scene-level artifacts—lens illumination patterns, vignetting, and color calibration—are essentially constant across images while this homogeneity is convenient for benchmarking sampling strategies, it can mask domain-shift sensitivity that would emerge once the model is deployed at hospitals operating different acquisition pipelines. A multi-center evaluation that includes commodity smartphone-based fundus cameras and at least one external publicly available dataset (e.g., Messidor, EyePACS, IDRiD) is required before any claim of clinical generality. We commit to this multi-center, external-dataset validation as the immediate next step, and we have correspondingly softened the clinical generality language in the Abstract and Conclusion so that it does not extend beyond the single-center NMC results reported here. We treat this as the principal external-validity threat and an immediate item of future work.

As a first step toward external contextualization, we additionally evaluated our multi-class YOLOv8x detector (trained jointly on IDRiD and NMC data for MA, HE, and EX, plus soft exudates and optic disc) on IDRiD’s own held-out validation split, obtaining an overall mAP@50 of 0.44 (per-class AP50: MA 0.207, HE 0.288, EX 0.224, SE 0.498, OD 0.995; Figure 7), compared with mAP@50 =0.40 at R7 for our single-class active-learning pipeline on the NMC pool (Table 4). The two numbers are only approximately comparable—IDRiD and NMC differ in camera, population, and label granularity (multi-class vs. unified lesion-presence)—but they place our NMC results in the same broad range as a directly evaluated IDRiD baseline rather than in a different regime entirely. A protocol-matched, head-to-head benchmark against published IDRiD and DDR leaderboard results is left for future work.

Second, our four active-learning sampling strategies (Average Confidence, Random, Hybrid-Diversity, MC Dropout) are evaluated under a fixed query budget of 50 images per round, an empirically chosen value matched to the throughput of two ophthalmologists at NMC. We deliberately did not co-optimize the per-round budget jointly with the sampling strategy. The convergence patterns observed in Figure 3 suggest that an automated mechanism that dynamically adjusts the per-round budget and switches sampling strategies as the model matures—for instance, Random in the cold-start regime and MC Dropout in later rounds—could further improve the cost–accuracy frontier. Designing such an adaptive controller is an open problem that we leave for subsequent work.

We consolidate here the justification for our key hyperparameter choices, which were described as empirical but not systematically validated. The per-round acquisition budget k=50 was set to match the realistic per-round throughput of two ophthalmologists at NMC within the deployment window, as noted above. The number of MC Dropout forward passes T=10 follows the standard range used by Gal and Ghahramani [25] for cheap epistemic-uncertainty estimation and was not separately tuned, since increasing *T* trades off directly against the per-round wall-clock budget (Section 3.3). The Hybrid-Diversity uncertainty fraction ρ=0.7 and the MC Dropout convex-combination weight λ=0.7 (set equal to ρ by design) were both chosen empirically during preliminary tests to balance uncertainty exploration against data diversity within our limited per-round batch size, rather than via a systematic sweep. We flag the non-exhaustive, empirical nature of the ρ/λ choice explicitly as a limitation; a sensitivity analysis over ρ,λ∈{0.5,0.6,0.7,0.8} is a natural and inexpensive extension for future work.

Third, the labeling-time experiment in Section 4.2.2 shows no statistically significant difference in overall per-image labeling time between AI-assisted and manual annotation (p=0.52), and a statistically significant increase in confirmed lesion detections (p=0.0058), concentrated in microaneurysms. While the mean mAP@50 of 0.40 represents a moderate performance level, our clinical HITL analysis confirms the system’s utility in reallocating cognitive effort toward identifying clinically important microvascular lesions rather than purely re-drawing boxes from scratch. The cumulative effect at the scale of a full dataset will be subject to nonlinear factors that our short-form experiment cannot capture: clinician fatigue over multi-hour sessions, inter-observer variance on hard cases, and the learning curve. As the subgroup sample sizes for lesion-burden stratification are small (n=18 and n=13), the observed time trends should be treated as hypothesis-generating rather than confirmatory. A longitudinal study with a larger paired sample is needed to confirm both the time-neutrality finding and the detection-quality gain reported here.

Finally, the proposed pipeline assumes that an AI pre-label is always presented as the starting point of expert review. This design choice carries a known automation-bias risk: clinicians may anchor on a confident-but-wrong AI prediction more readily than they would correct a blank canvas. Our current HITL platform mitigates this through keyboard-shortcut-driven box deletion and forced two-clinician review on flagged cases, but a principled study of the false-confirmation rate—in particular for microaneurysms, where lesions are visually subtle—is a worthwhile direction for safety-oriented future work. Relatedly, the round-by-round active-learning comparison in Section 4.2.1 currently reports only aggregate detection metrics; instrumenting the pipeline for multi-class labels so that per-strategy, per-round AP-MA/AP-HE/AP-EX can be reported directly (rather than via the separate multi-class detector evaluated in Section 4.2.2) is a concrete extension we leave for future work.

## 6. Conclusions

To address the bottleneck problem of high-cost medical image labeling, this study proposes an active learning pipeline based on human-AI interaction. Experimental results using real-world clinical data demonstrate that this system does not significantly change overall annotation time (p=0.52) but significantly increases the number of confirmed lesion detections under AI assistance (p=0.0058), predominantly for microaneurysms. This confirms that high-performance diagnostic models can be effectively trained even under a limited annotation budget and redefines the system’s value proposition from simple time savings to annotation quality enhancement.

However, the scale of the current dataset remains limited, necessitating further expert collaboration and data acquisition to reflect a more diverse patient population and varied imaging conditions. Furthermore, introducing an automated tuning mechanism to dynamically optimize the sampling strategy and the HITL cycle during the active learning process remains a task for future work. Additionally, because the dataset itself is acquired using the same fundus camera within the NMC, identical artifacts may exist across the images, potentially posing challenges to the generalization performance of the model.

Future research aims to extend the efficient data construction process proposed in this study to other high-cost medical imaging modalities, such as CT and MRI, to verify its applicability and versatility. This approach is expected to accelerate the clinical integration of AI-based medical imaging technologies, ultimately contributing to simultaneous improvements in healthcare accessibility and diagnostic efficiency.

## Figures and Tables

**Table 1:** Distinction matrix: Virtuous Cycle vs. the closest prior work along the DR-AI, active-learning, AL-for-detection, and HITL axes. “AL?” marks active-learning sample selection; “HITL?” marks expert review and correction inside the training loop; “Real clinician?” indicates whether actual clinicians (rather than simulated labelers) generated the labels used in the reported results; “Deployed?” indicates whether the system has been integrated into a hospital workflow at the time of publication.

System	Task	AL?	HITL?	Real Clinician?	Deployed?
Gulshan et al. [27]	DR cls.	no	no	—	offline
Ting et al. [28]	DR cls.	no	no	—	screening prog.
Abràmoff et al. [20]	DR cls.	no	no	—	FDA-cleared
Wolf et al. [5]	DR cls.	no	no	—	RCT trial
Dai et al. [6]	DR risk	no	no	—	internal
Heydon et al. [21]	DR cls.	no	no	—	CE-marked
Dow et al. [7]	DR cls.	no	2-stage	yes	trial
Paul et al. [22]	DR cls.	4 strats	no	—	offline
Yuan et al. [37]	generic det.	MI-AOD	no	—	offline
Yu et al. [38]	generic det.	CALD	no	—	offline
Wang et al. [12]	survey	review	—	—	—
Vásquez-Venegas et al. [15]	COVID-19 CT	no	yes	yes	offline
Ma et al. [23]	generic cls.	curriculum	no	—	offline
Virtuous Cycle (this work)	DR det.	4 (adapt.)	cyclical	yes	NMC

**Table 4:** Per-strategy detection metrics (mean ± std across three independent random seeds) across active-learning rounds on the NMC dataset. R_*i*_ denotes the *i*-th active-learning round; the labeled pool grows from 81 images at R0 to 431 at R7 and 481 at R8. Since R8 utilizes the entire available (non-test) NMC pool, all four strategies necessarily converge to the same final model; we therefore report this converged performance as the reference baseline. Bold = best per metric at R7 (the last round at which the strategies still differ).

Strategy	R0	R1	R3	R5	R7	R8 (481)
mAP@50						
Random	0.14 ± 0.09	0.25 ± 0.02	0.33 ± 0.03	0.34 ± 0.02	0.36 ± 0.05	0.40 ± 0.07
Avg. Confidence	0.14 ± 0.09	0.12 ± 0.09	0.28 ± 0.05	0.32 ± 0.05	0.37 ± 0.05	0.40 ± 0.07
Hybrid	0.14 ± 0.09	0.18 ± 0.06	0.31 ± 0.05	0.35 ± 0.06	**0.40 ± 0.08**	0.40 ± 0.07
MC Dropout	0.14 ± 0.09	0.12 ± 0.08	0.31 ± 0.06	0.34 ± 0.07	0.38 ± 0.08	0.40 ± 0.07
mAP@50–95						
Random	0.05 ± 0.03	0.09 ± 0.01	0.13 ± 0.02	0.13 ± 0.02	0.14 ± 0.03	0.16 ± 0.04
Avg. Confidence	0.05 ± 0.03	0.04 ± 0.03	0.11 ± 0.02	0.12 ± 0.02	0.15 ± 0.03	0.16 ± 0.04
Hybrid	0.05 ± 0.03	0.06 ± 0.03	0.12 ± 0.03	0.14 ± 0.03	**0.15 ± 0.03**	0.16 ± 0.04
MC Dropout	0.05 ± 0.03	0.04 ± 0.03	0.12 ± 0.03	0.14 ± 0.04	0.15 ± 0.04	0.16 ± 0.04
Precision						
Random	0.40 ± 0.00	0.42 ± 0.05	0.50 ± 0.09	0.50 ± 0.05	0.51 ± 0.04	0.55 ± 0.01
Avg. Confidence	0.40 ± 0.00	0.26 ± 0.18	0.44 ± 0.04	0.47 ± 0.04	0.55 ± 0.04	0.55 ± 0.01
Hybrid	0.40 ± 0.00	0.44 ± 0.04	0.48 ± 0.03	0.51 ± 0.02	**0.55 ± 0.01**	0.55 ± 0.01
MC Dropout	0.40 ± 0.00	0.26 ± 0.19	0.46 ± 0.03	0.51 ± 0.03	0.51 ± 0.01	0.55 ± 0.01
Recall						
Random	0.17 ± 0.10	0.30 ± 0.04	0.37 ± 0.06	0.36 ± 0.03	0.39 ± 0.06	0.43 ± 0.07
Avg. Confidence	0.17 ± 0.10	0.16 ± 0.11	0.33 ± 0.07	0.35 ± 0.07	0.38 ± 0.06	0.43 ± 0.07
Hybrid	0.17 ± 0.10	0.19 ± 0.08	0.35 ± 0.06	0.40 ± 0.07	**0.41 ± 0.08**	0.43 ± 0.07
MC Dropout	0.17 ± 0.10	0.15 ± 0.10	0.35 ± 0.07	0.37 ± 0.09	0.40 ± 0.07	0.43 ± 0.07

## Data Availability

The NMC diabetic retinopathy fundus image dataset analyzed in this study is not publicly available due to patient-privacy and institutional policies. Anonymized data are available from the corresponding author on reasonable request and subject to approval by the NMC Institutional Review Board. The pre-trained YOLOv8x backbone weights are publicly available from the Ultralytics project [24]. To ensure algorithmic reproducibility without compromising patient privacy, a clean implementation of the proposed active learning pipeline and the four sampling strategies is available at: https://github.com/bigbases/VIRTUOUS_CYCLE (accessed on 25 June 2026).

## Abbreviations

The following abbreviations are used in this manuscript:

AI	Artificial Intelligence
AL	Active Learning
CE	CE marking (Conformité Européenne)
DR	Diabetic Retinopathy
EX	Exudates
HE	Hemorrhages
HITL	Human-in-the-Loop
IRB	Institutional Review Board
MA	Microaneurysms
mAP	mean Average Precision
MC	Monte Carlo
NMC	National Medical Center
OD	Optic Disc
SE	Soft Exudates
UQ	Uncertainty Quantification
WHO	World Health Organization
YOLO	You Only Look Once
